# *In Vitro* Activity of Eravacycline against Gram-Positive Bacteria Isolated in Clinical Laboratories Worldwide from 2013 to 2017

**DOI:** 10.1128/AAC.01715-19

**Published:** 2020-02-21

**Authors:** Ian Morrissey, Stephen Hawser, Sibylle H. Lob, James A. Karlowsky, Matteo Bassetti, G. Ralph Corey, Melanie Olesky, Joseph Newman, Corey Fyfe

**Affiliations:** a IHMA Europe Sàrl, Monthey, Switzerland; b International Health Management Associates, Inc., Schaumburg, Illinois, USA; c Department of Medical Microbiology and Infectious Diseases, Max Rady College of Medicine, University of Manitoba, Winnipeg, Manitoba, Canada; d Infectious Diseases Clinic, Department of Medicine, University of Udine, Udine, Italy; e Duke University Medical Center, Durham, North Carolina, USA; f Tetraphase Pharmaceuticals, Watertown, Massachusetts, USA

**Keywords:** eravacycline, Gram positive, MRSA, VRE, streptococci, *Streptococcus*

## Abstract

Eravacycline is a novel, fully synthetic fluorocycline antibiotic being developed for the treatment of serious infections, including those caused by resistant Gram-positive pathogens. Here, we evaluated the *in vitro* activities of eravacycline and comparator antimicrobial agents against a recent global collection of frequently encountered clinical isolates of Gram-positive bacteria. The CLSI broth microdilution method was used to determine *in vitro* MIC data for isolates of *Enterococcus* spp. (*n* = 2,807), *Staphylococcus* spp. (*n* = 4,331), and *Streptococcus* spp. (*n* = 3,373) isolated primarily from respiratory, intra-abdominal, urinary, and skin specimens by clinical laboratories in 37 countries on three continents from 2013 to 2017. Susceptibilities were interpreted using both CLSI and EUCAST breakpoints. There were no substantive differences (a >1-doubling-dilution increase or decrease) in eravacycline MIC_90_ values for different species/organism groups over time or by region. Eravacycline showed MIC_50_ and MIC_90_ results of 0.06 and 0.12 μg/ml, respectively, when tested against Staphylococcus aureus, regardless of methicillin susceptibility. The MIC_90_ values of eravacycline for Staphylococcus epidermidis and Staphylococcus haemolyticus were equal (0.5 μg/ml). The eravacycline MIC_90_s for Enterococcus faecalis and Enterococcus faecium were 0.06 μg/ml and were within 1 doubling dilution regardless of the vancomycin susceptibility profile. Eravacycline exhibited MIC_90_ results of ≤0.06 μg/ml when tested against Streptococcus pneumoniae and beta-hemolytic and viridans group streptococcal isolates. In this surveillance study, eravacycline demonstrated potent *in vitro* activity against frequently isolated clinical isolates of Gram-positive bacteria (*Enterococcus*, *Staphylococcus*, and *Streptococcus* spp.), including isolates collected over a 5-year period (2013 to 2017), underscoring its potential benefit in the treatment of infections caused by common Gram-positive pathogens.

## Abstract (precis)

Eravacycline is a novel, fully synthetic fluorocycline antibiotic being developed for the treatment of serious infections, including those caused by resistant Gram-positive pathogens. Here, we evaluated the *in vitro* activities of eravacycline and comparator antimicrobial agents against a recent global collection of frequently encountered clinical isolates of Gram-positive bacteria. The CLSI broth microdilution method was used to determine *in vitro* MIC data for isolates of *Enterococcus* spp.

## INTRODUCTION

Multidrug-resistant (MDR) Gram-positive organisms are major human pathogens, causing both health care-associated and community-acquired infections. Clinically important antimicrobial-resistant Gram-positive pathogens include methicillin-resistant Staphylococcus aureus (MRSA), vancomycin-resistant enterococci (VRE), and Streptococcus pneumoniae. In fact, all three have recently been highlighted among the Gram-positive pathogens classified as serious or high public health threats by the Centers for Disease Control and Prevention (CDC) (1) and the World Health Organization (WHO) (2).

Eravacycline is a novel, fully synthetic fluorocycline antibiotic recently approved by U.S. Food and Drug Administration (FDA) and the European Medicines Agency (EMA) for the treatment of complicated intra-abdominal infections (cIAI), including those caused by MDR pathogens (3, 4; https://clinicaltrials.gov/ct2/show/NCT01844856). Additionally, eravacycline has been demonstrated to have *in vivo* efficacy as a treatment in murine models of systemic, thigh, and lung infection and pyelonephritis (4, 6, 7).

Eravacycline is comprised of a tetracycline core with two novel modifications: a fluorine atom at the C-7 position and a pyrrolidinoacetamido group at the C-9 position, both of which are on the D ring (4, 8). These novel modifications confer enhanced *in vitro* activity compared to that of other tetracyclines against resistant Gram-negative and Gram-positive bacteria, and the pyrrolidinoacetamido group allows for increased ribosomal binding and steric hindrance to avoid ribosome protection-based tetracycline resistance.

Eravacycline inhibits bacterial protein synthesis (i.e., acyl-tRNA transfer) by binding to the 30S ribosomal subunit (9). Eravacycline demonstrates potent broad-spectrum activity against Gram-positive cocci and Gram-negative bacilli (except Pseudomonas aeruginosa and *Burkholderia* spp.), including anaerobes, as well as atypical bacterial pathogens and Neisseria gonorrhoeae (3, 10–15), and does not exhibit a loss of antibacterial activity against isolates expressing tetracycline ribosomal protection genes or most tetracycline efflux resistance genes (9, 10, 13).

The objective of the current study was to determine the *in vitro* activity of eravacycline relative to that of other antimicrobial agents using a representative global collection of clinical isolates of Gram-positive bacteria.

## RESULTS AND DISCUSSION

A total of 10,511 Gram-positive aerobic isolates collected between 2013 and 2017 were included in this study. The MIC distributions and the cumulative percentage of selected isolates of Gram-positive bacteria tested inhibited by eravacycline are shown in Table 1. The MIC_90_ of eravacycline for isolates of S. aureus was 0.12 μg/ml irrespective of whether the isolates were MRSA or methicillin-susceptible S. aureus (MSSA). The eravacycline MIC_90_ values for the coagulase-negative staphylococci Staphylococcus epidermidis and Staphylococcus haemolyticus, including the methicillin-resistant subsets, were ≤0.5 μg/ml. The eravacycline MIC_90_ for Enterococcus faecalis was 0.06 μg/ml, with a 1-doubling-dilution shift being seen for vancomycin-resistant E. faecalis. The eravacycline MIC_90_ for Enterococcus faecium was 0.06 μg/ml, regardless of its vancomycin susceptibility. Eravacycline exhibited MIC_90_ results of ≤0.06 μg/ml when tested against beta-hemolytic and viridans group streptococci as well as an MIC_90_ of 0.015 μg/ml for Streptococcus pneumoniae.

**TABLE 1:** Cumulative percentage of clinical isolates of staphylococci, enterococci, and streptococci tested from 2013 to 2017 inhibited by eravacycline, by MIC

Organism^*g*^	No. of isolates	Cumulative % of isolates inhibited by the following eravacycline MIC (μg/ml)^*a*^:											
		≤0.001	0.002	0.004	0.008	0.015	0.03	0.06	0.12	0.25	0.5	1	2
S. aureus^*b*^	2,588				0.4	3.8	33.4	84.5	94.8	97.6	99.4	100	
S. aureus, MR^*b*^	1,304				0.2	4.1	32.8	80.8	91.6	95.5	98.8	100	
S. aureus, MS^*b*^	1,284				0.5	3.5	34.0	88.3	98.0	99.8	100		
S. epidermidis^*b*^	1,012				1.4	11.5	28.2	45.7	63.4	84.6	97.1	99.8	100
S. epidermidis, MR^*b*^^,^^*c*^	480				1.9	10.8	30.8	43.8	66.7	90.4	97.9	99.8	100
S. epidermidis, MS^*b*^^,^^*c*^	255				2.0	24.3	47.8	59.6	75.7	96.9	99.6	99.6	100
S. haemolyticus^*b*^	731				1.2	13.5	34.6	45.6	64.8	89.9	96.0	98.8	100
S. haemolyticus, MR^*b*^^,^^*c*^	440				0.5	11.4	30.9	36.8	60.2	90.0	95.2	98.0	100
S. haemolyticus, MS^*b*^^,^^*c*^	134				5.2	35.8	76.9	85.8	94.8	97.8	99.3	100	
E. faecalis	1,586				0.2	2.6	28.9	94.5	99.4	99.7	100		
E. faecalis, VR^*d*^	59						23.7	89.8	98.3	100			
E. faecalis, VS	1,505				0.2	2.7	29.4	94.8	99.5	99.7	100		
E. faecium	1,221				0.6	4.3	60.2	95.0	97.7	99.1	99.8	100	
E. faecium, VR^*d*^	510				0.6	3.7	54.9	93.1	96.1	98.0	99.6	100	
E. faecium, VS	702				0.6	4.6	63.8	96.3	98.9	99.9	100		
S. pneumoniae^*e*^	596	0.8	2.0	14.8	73.0	97.8	100						
S. agalactiae	1,239				0.7	13.4	70.5	98.0	99.8	100			
S. pyogenes	1,192			0.2	3.6	47.8	96.0	100					
S. anginosus group^*f*^	346	5.2	5.5	8.7	19.9	46.5	86.4	99.1	100				

a The MIC_90_ is shaded gray.

b For staphylococci, the lowest dilution of eravacycline tested was 0.008 μg/ml.

c Defined using oxacillin MICs, which were available only for 2015 to 2017.

d Defined using CLSI breakpoint criteria.

e Collected only in 2013 to 2014 and 2017.

f The S. anginosus group (*n* = 346) includes S. anginosus (*n* = 302), S. constellatus (*n* = 36), S. intermedius (*n* = 7), and S. intermedius/S. milleri (*n* = 1).

g MR, methicillin resistant; MS, methicillin susceptible; VR, vancomycin resistant; VS, vancomycin susceptible.

Tables 2, 3, and 4 provide details on the *in vitro* activities of eravacycline and the comparator agents against staphylococci, enterococci, and streptococci, respectively, including percent susceptibility according to the CLSI and EUCAST breakpoints. The highest rates of nonsusceptibility in MRSA were reported for azithromycin, clindamycin, and levofloxacin (75.9%, 38.3%, and 65.9%, respectively, by CLSI criteria), while resistance rates were <1% for linezolid, daptomycin, and vancomycin (Table 2). For compounds of the tetracycline class, tigecycline and minocycline, resistance rates were approximately 2 to 12% across FDA/CLSI and EUCAST breakpoints. Comparatively, due to overall lower breakpoints for eravacycline, the nonsusceptible rate was nearly 20% by the FDA criteria and 4.5% by the EUCAST criteria, but the MIC_90_ value of eravacycline was 2-fold lower than that of tigecycline. Similarly, for E. faecalis the nonsusceptibility rates to linezolid and daptomycin were <1% and 5.6%, respectively, while the rates were 2% and 53%, respectively, for E. faecium (Table 3). Vancomycin retained activity against E. faecalis, with a resistance rate of 4.9%, but it was generally ineffective against E. faecium, in which the rate of resistance exceeded 40%. Both species of enterococci were resistant to minocycline, with nonsusceptibility rates ranging from 49 to 72%. While eravacycline and tigecycline nonsusceptibility rates were about 1 to 5%, the MIC_90_ of tigecycline was 2 doubling dilutions higher than that of eravacycline. Notably, the rates of resistance for the comparators in this study were similar to those seen in other global surveillance studies (16, 17).

**TABLE 2:** *In vitro* activity of eravacycline and comparator agents against staphylococci, cumulative 2013 to 2017 data^*d*^

Organism	Drug	No. of isolates	MIC (μg/ml)			% susceptible	
			50%	90%	Range	CLSI	EUCAST
S. aureus	Eravacycline	2,588	0.06	0.12	≤0.008 to 1	84.5^*a*^	97.6
	Amoxicillin-clavulanate	2,588	>1	>1	≤0.12 to >1	NA	NA
	Azithromycin	2,588	>4	>4	≤0.25 to >4	49.3	47.8
	Ceftaroline	1,076	0.5	1	≤0.06 to >4	94.1	94.1
	Ceftriaxone	980	16	>64	1 to >64	NA	NA
	Clindamycin	1,608	0.12	>2	≤0.03 to >2	78.3	78.2
	Daptomycin	2,588	0.5	1	≤0.06 to 4	99.8	99.8
	Gentamicin	532	0.25	0.5	≤0.06 to >8	92.5	92.1
	Levofloxacin	2,588	0.25	>4	≤0.03 to >4	62.1	62.1
	Linezolid	2,588	2	2	≤0.5 to 2	100	100
	Minocycline	2,587	0.12	0.25	≤0.06 to >8	95.2	93.2
	Oxacillin	1,608	>2	>2	≤0.06 to >2	49.6	49.6
	Penicillin	2,588	>2	>2	≤0.12 to >2	13.4	13.4
	Tetracycline	2,588	0.25	>16	≤0.06 to >16	87.6	86.1
	Tigecycline	2,588	0.12	0.25	0.03 to 2	98.6^*a*^	98.6
	Vancomycin	1,608	1	1	≤0.25 to 2	100	100
S. aureus, MR	Eravacycline	1,304	0.06	0.12	≤0.008 to 1	80.8^*a*^	95.5
	Amoxicillin-clavulanate	1,304	>1	>1	0.5 to >1	NA	NA
	Azithromycin	1,304	>4	>4	≤0.25 to >4	24.1	23.1
	Ceftaroline	548	1	2	0.12 to >4	88.3	88.3
	Ceftriaxone	493	>64	>64	4 to >64	NA	NA
	Clindamycin	811	0.12	>2	≤0.03 to >2	61.7	61.7
	Daptomycin	1,304	0.5	1	≤0.06 to 4	99.6	99.6
	Gentamicin	263	0.25	>8	0.12 to >8	85.6	85.2
	Levofloxacin	1,304	>4	>4	0.06 to >4	34.1	34.1
	Linezolid	1,304	2	2	≤0.5 to 2	100	100
	Minocycline	1,303	0.12	4	≤0.06 to >8	91.8	88.2
	Oxacillin	811	>2	>2	1 to >2	0.1	0.1
	Penicillin	1,304	>2	>2	≤0.12 to >2	0.2	0.2
	Tetracycline	1,304	0.25	>16	≤0.06 to >16	82.0	79.8
	Tigecycline	1,304	0.12	0.25	0.03 to 2	97.5^*a*^	97.5
	Vancomycin	811	1	1	≤0.25 to 2	100	100
S. aureus, MS	Eravacycline	1,284	0.06	0.12	≤0.008 to 0.5	88.3^*a*^	99.8
	Amoxicillin-clavulanate	1,284	1	>1	≤0.12 to >1	NA	NA
	Azithromycin	1284	1	>4	≤0.25 to >4	74.9	73.0
	Ceftaroline	528	0.25	0.25	≤0.06 to 0.5	100	100
	Ceftriaxone	487	4	8	1 to >64	NA	NA
	Clindamycin	797	0.06	0.12	≤0.03 to >2	95.2	95.0
	Daptomycin	1,284	0.5	0.5	0.12 to 2	99.9	99.9
	Gentamicin	269	0.25	0.5	≤0.06 to >8	99.3	98.9
	Levofloxacin	1,284	0.25	1	≤0.03 to >4	90.5	90.5
	Linezolid	1,284	2	2	≤0.5 to 2	100	100
	Minocycline	1,284	0.12	0.12	≤0.06 to >8	98.7	98.3
	Oxacillin	797	0.25	0.5	≤0.06 to >2	99.9	99.9
	Penicillin	1,284	2	>2	≤0.12 to >2	26.8	26.8
	Tetracycline	1,284	0.25	0.5	≤0.06 to >16	93.4	92.5
	Tigecycline	1,284	0.12	0.25	0.03 to 1	99.7^*a*^	99.7
	Vancomycin	797	1	1	≤0.25 to 2	100	100
S. epidermidis	Eravacycline	1,012	0.12	0.5	≤0.008 to 2	45.7^*a*^	84.6^*b*^
	Amoxicillin-clavulanate	1,012	>1	>1	≤0.12 to >1	NA	NA
	Azithromycin	1,012	>4	>4	≤0.25 to >4	37.2	36.9
	Ceftaroline	529	0.25	0.5	≤0.06 to >4	NA	NA
	Ceftriaxone	277	16	>64	≤0.5 to >64	NA	NA
	Clindamycin	735	0.06	>2	≤0.03 to >2	72.0	68.6
	Daptomycin	1,012	0.5	1	≤0.06 to 4	99.5	99.5
	Gentamicin	206	0.12	>8	≤0.06 to >8	62.6	55.8
	Levofloxacin	1,012	2	>4	0.06 to >4	45.6	45.6
	Linezolid	1,012	1	2	≤0.5 to >8	98.5	98.5
	Minocycline	1,012	0.12	0.5	≤0.06 to >8	99.6	98.7
	Oxacillin	735	2	>2	≤0.06 to >2	34.7	34.7
	Penicillin	1,012	>2	>2	≤0.12 to >2	11.1	NA
	Tetracycline	1,012	1	>16	≤0.06 to >16	85.7	66.5
	Tigecycline	1,012	0.12	0.5	≤0.015 to 1	97.5^*a*^	97.5
	Vancomycin	735	1	2	0.5 to 2	100	100
S. epidermidis, MR^*c*^	Eravacycline	480	0.12	0.25	≤0.008 to 2	43.8^*a*^	90.4^*b*^
	Amoxicillin-clavulanate	480	>1	>1	≤0.12 to >1	NA	NA
	Azithromycin	480	>4	>4	≤0.25 to >4	30.6	30.4
	Ceftaroline	326	0.5	1	≤0.06 to >4	NA	NA
	Clindamycin	480	0.12	>2	≤0.03 to >2	61.7	57.7
	Daptomycin	480	0.5	0.5	≤0.06 to 1	100	100
	Gentamicin	154	4	>8	≤0.06 to >8	52.0	44.2
	Levofloxacin	480	4	>4	0.06 to >4	26.3	26.3
	Linezolid	480	≤0.5	1	≤0.5 to >4	97.7	97.7
	Minocycline	480	0.12	0.5	≤0.06 to >8	99.4	98.8
	Oxacillin	480	>2	>2	0.5 to >2	0.0	0.0
	Penicillin	480	>2	>2	≤0.12 to >2	1.3	NA
	Tetracycline	480	1	>16	≤0.06 to >16	84.8	70.2
	Tigecycline	480	0.12	0.25	≤0.015 to 1	99.0^*a*^	99.0
	Vancomycin	480	1	2	0.5 to 2	100	100
S. epidermidis, MS^*c*^	Eravacycline	255	0.06	0.25	≤0.008 to 2	59.6^*a*^	96.9^*b*^
	Amoxicillin-clavulanate	255	0.25	0.5	≤0.12 to >1	NA	NA
	Azithromycin	255	0.5	>4	≤0.25 to >4	53.3	52.9
	Ceftaroline	203	≤0.06	0.12	≤0.06 to 0.5	NA	NA
	Clindamycin	255	0.06	0.5	≤0.03 to >2	91.4	89.0
	Daptomycin	255	0.5	0.5	≤0.06 to 1	100	100
	Gentamicin	52	≤0.06	0.25	≤0.06 to >8	94.2	90.4
	Levofloxacin	255	0.25	>4	0.06 to >4	81.6	81.6
	Linezolid	255	≤0.5	1	≤0.5 to >4	99.6	99.6
	Minocycline	255	0.12	0.25	≤0.06 to 4	100	99.6
	Oxacillin	255	0.12	0.12	≤0.06 to 0.25	100	100
	Penicillin	255	0.5	2	≤0.12 to >2	29.4	NA
	Tetracycline	255	0.5	4	≤0.06 to >16	90.6	80.8
	Tigecycline	255	0.12	0.25	0.03 to 1	99.6^*a*^	99.6
	Vancomycin	255	1	2	0.5 to 2	100	100
S. haemolyticus	Eravacycline	731	0.12	0.5	≤0.008 to 2	45.6^*a*^	89.9^*b*^
	Amoxicillin-clavulanate	731	>1	>1	≤0.12 to >1	NA	NA
	Azithromycin	731	>4	>4	≤0.25 to >4	20.4	20.1
	Ceftaroline	426	1	2	≤0.06 to >4	NA	NA
	Ceftriaxone	157	>64	>64	1 to >64	NA	NA
	Clindamycin	574	0.06	>2	≤0.03 to >2	80.0	79.6
	Daptomycin	731	0.5	0.5	0.12 to 2	99.9	99.9
	Gentamicin	148	8	>8	≤0.06 to >8	39.9	29.1
	Levofloxacin	731	>4	>4	0.06 to >4	28.9	28.9
	Linezolid	732	1	2	≤0.5 to >8	99.9	99.9
	Minocycline	731	0.12	0.25	≤0.06 to 8	99.7	98.1
	Oxacillin	574	>2	>2	≤0.06 to >2	23.3	23.3
	Penicillin	731	>2	>2	≤0.12 to >2	17.2	NA
	Tetracycline	731	1	>16	≤0.06 to >16	81.0	69.8
	Tigecycline	731	0.25	0.5	0.03 to 2	95.9^*a*^	95.9
	Vancomycin	574	1	2	≤0.25 to 4	100	100
S. haemolyticus, MR^*c*^	Eravacycline	440	0.12	0.25	≤0.008 to 2	36.8^*a*^	90.0^*b*^
	Amoxicillin-clavulanate	440	>1	>1	≤0.12 to >1	NA	NA
	Azithromycin	440	>4	>4	≤0.25 to >4	9.8	9.8
	Ceftaroline	317	2	2	0.12 to >4	NA	NA
	Clindamycin	440	0.06	>2	≤0.03 to >2	75.0	74.6
	Daptomycin	440	0.5	0.5	0.12 to 1	100	100
	Gentamicin	123	>8	>8	≤0.06 to >8	30.1	17.9
	Levofloxacin	440	>4	>4	0.06 to >4	13.0	13.0
	Linezolid	441	1	1	≤0.5 to 2	100	100
	Minocycline	440	0.25	0.25	≤0.06 to 8	99.8	97.5
	Oxacillin	440	>2	>2	0.5 to >2	0.0	0.0
	Penicillin	440	>2	>2	≤0.12 to >2	1.6	NA
	Tetracycline	440	1	>16	0.12 to >16	82.3	71.8
	Tigecycline	440	0.25	0.5	0.06 to 2	95.5^*a*^	95.5
	Vancomycin	440	1	2	≤0.25 to 4	100	100
S. haemolyticus, MS^*c*^	Eravacycline	134	0.03	0.12	≤0.008 to 1	85.8^*a*^	97.8^*b*^
	Amoxicillin-clavulanate	134	≤0.12	0.5	≤0.12 to >1	NA	NA
	Azithromycin	134	0.5	>4	≤0.25 to >4	54.5	53.0
	Ceftaroline	109	0.25	0.25	≤0.06 to 2	NA	NA
	Clindamycin	134	0.06	0.25	≤0.03 to >2	96.3	96.3
	Daptomycin	134	0.25	0.5	0.12 to 0.5	100	100
	Gentamicin	25	≤0.06	8	≤0.06 to >8	88.0	84.0
	Levofloxacin	134	0.12	4	0.06 to >4	85.1	85.1
	Linezolid	134	≤0.5	1	≤0.5 to 2	100	100
	Minocycline	134	≤0.06	0.25	≤0.06 to 0.5	100	100
	Oxacillin	134	0.12	0.25	≤0.06 to 0.25	100	100
	Penicillin	134	≤0.12	1	≤0.12 to >2	74.6	NA
	Tetracycline	134	0.25	>16	≤0.06 to >16	79.1	78.4
	Tigecycline	134	0.12	0.25	0.06 to 0.5	100^*a*^	100
	Vancomycin	134	0.5	1	≤0.25 to 2	100	100

a U.S. Food and Drug Administration (FDA) MIC interpretative breakpoints were used in place of CLSI MIC breakpoints for eravacycline (≤0.06 μg/ml) and tigecycline (≤0.5 μg/ml) (21), as none currently exist. FDA eravacycline and tigecycline breakpoints for S. aureus were applied to the tested coagulase-negative *Staphylococcus* species.

b EUCAST eravacycline breakpoints for S. aureus (≤0.25 μg/ml) were applied to the coagulase-negative *Staphylococcus* species tested.

c Defined using oxacillin MICs, which were available only for 2015 to 2017.

d MR, methicillin resistant; MS, methicillin susceptible; NA, MIC breakpoint not available.

**TABLE 3:** *In vitro* activity of eravacycline and comparator agents against enterococci, cumulative 2013 to 2017 data^*c*^

Organism	Drug	No. of isolates	MIC (μg/ml)			% susceptible	
			50%	90%	Range	CLSI	EUCAST
E. faecalis	Eravacycline	1,586	0.06	0.06	0.008 to 0.5	94.5^*a*^	99.4
	Amoxicillin-clavulanate	1,586	1	1	≤0.12 to >1	NA	99.9^*b*^
	Ampicillin	1,085	1	2	≤0.25 to >8	99.3	99.3
	Azithromycin	501	>8	>8	≤0.12 to >8	NA	NA
	Ceftriaxone	501	>64	>64	≤0.5 to >64	NA	NA
	Daptomycin	1,586	1	2	≤0.06 to 8	94.4	NA
	Levofloxacin	1,586	1	>8	≤0.03 to >8	69.1	69.5
	Linezolid	1,586	2	2	≤0.5 to >4	99.4	99.9
	Minocycline	1,586	>8	>8	≤0.03 to >8	27.8	NA
	Penicillin	1,586	2	4	≤0.12 to >8	97.6	NA
	Tetracycline	1,586	>32	>32	≤0.06 to >32	22.3	NA
	Tigecycline	1,586	0.12	0.25	≤0.015 to 8	94.8^*a*^	94.8
	Vancomycin	1,582	1	2	0.12 to >32	95.1	95.1
E. faecalis, VR	Eravacycline	59	0.06	0.12	0.03 to 0.25	89.8^*a*^	98.3
	Amoxicillin-clavulanate	59	1	>1	0.5 to >1	NA	98.3^*b*^
	Ampicillin	34	1	2	1 to >8	97.1	97.1
	Azithromycin	25	>8	>8	2 to >8	NA	NA
	Ceftriaxone	25	>64	>64	4 to >64	NA	NA
	Daptomycin	59	1	2	0.5 to 4	96.6	NA
	Levofloxacin	59	>8	>8	0.5 to >8	5.1	5.1
	Linezolid	59	1	2	≤0.5 to 2	100	100
	Minocycline	59	>8	>8	0.06 to >8	15.3	NA
	Penicillin	59	4	8	1 to >8	96.6	NA
	Tetracycline	59	>32	>32	0.25 to >32	8.5	NA
	Tigecycline	59	0.12	0.25	0.06 to 1	94.9^*a*^	94.9
	Vancomycin	59	>16	>32	>16 to >32	0.0	0.0
E. faecalis, VS	Eravacycline	1,505	0.06	0.06	0.008 to 0.5	94.8^*a*^	99.5
	Amoxicillin-clavulanate	1,505	1	1	≤0.12 to >1	NA	100^*b*^
	Ampicillin	1,046	1	2	≤0.25 to >8	99.3	99.3
	Azithromycin	459	>8	>8	≤0.12 to >8	NA	NA
	Ceftriaxone	459	>64	>64	≤0.5 to >64	NA	NA
	Daptomycin	1,505	1	2	≤0.06 to 8	94.3	NA
	Levofloxacin	1,505	1	>8	≤0.03 to >8	71.8	72.2
	Linezolid	1,505	2	2	≤0.5 to >4	99.5	99.9
	Minocycline	1,505	>8	>8	≤0.03 to >8	28.1	NA
	Penicillin	1,505	2	4	≤0.12 to >8	97.7	NA
	Tetracycline	1,505	>32	>32	≤0.06 to >32	22.6	NA
	Tigecycline	1,505	0.12	0.25	≤0.015 to 8	94.8^*a*^	94.8
	Vancomycin	1,505	1	2	0.12 to 4	100	100
E. faecium	Eravacycline	1,221	0.03	0.06	0.008 to 1	95.0^*a*^	97.7
	Amoxicillin-clavulanate	1,221	>1	>1	≤0.12 to >1	NA	67.2^*b*^
	Ampicillin	762	>8	>8	≤0.25 to >8	11.8	11.2
	Azithromycin	459	>8	>8	0.25 to >8	NA	NA
	Ceftriaxone	459	>64	>64	1 to >64	NA	NA
	Daptomycin	1,221	4	4	≤0.06 to >8	47.5	NA
	Levofloxacin	1,221	>8	>8	0.06 to >8	8.9	13.1
	Linezolid	1,221	2	2	≤0.5 to >8	98.1	99.4
	Minocycline	1,221	4	>8	≤0.03 to >8	50.9	NA
	Penicillin	1,221	>8	>8	≤0.06 to >8	10.4	NA
	Tetracycline	1,221	32	>32	≤0.06 to >32	40.8	NA
	Tigecycline	1,221	0.12	0.25	≤0.015 to 8	95.2^*a*^	95.2
	Vancomycin	1,219	1	>32	≤0.12 to >32	57.6	57.6
E. faecium, VR	Eravacycline	510	0.03	0.06	0.008 to 1	93.1^*a*^	96.1
	Amoxicillin-clavulanate	510	>1	>1	≤0.12 to >1	NA	51.4^*b*^
	Ampicillin	256	>8	>8	0.5 to >8	0.8	0.8
	Azithromycin	254	>8	>8	0.25 to >8	NA	NA
	Ceftriaxone	254	>64	>64	1 to >64	NA	NA
	Daptomycin	510	2	4	0.12 to 8	50.4	NA
	Levofloxacin	510	>8	>8	2 to >8	0.2	0.2
	Linezolid	510	2	2	≤0.5 to >8	98.0	98.8
	Minocycline	510	8	>8	≤0.03 to >8	42.8	NA
	Penicillin	510	>8	>8	0.5 to >8	1.0	NA
	Tetracycline	510	32	>32	0.12 to >32	26.1	NA
	Tigecycline	510	0.12	0.25	0.03 to 4	93.7^*a*^	93.7
	Vancomycin	510	>16	>32	>16 to >32	0.0	0.0
E. faecium, VS	Eravacycline	702	0.03	0.06	0.008 to 0.5	96.3^*a*^	98.9
	Amoxicillin-clavulanate	702	>1	>1	≤0.12 to >1	NA	79.2^*b*^
	Ampicillin	504	>8	>8	≤0.25 to >8	17.5	16.5
	Azithromycin	198	>8	>8	0.25 to >8	NA	NA
	Ceftriaxone	198	>64	>64	1 to >64	NA	NA
	Daptomycin	702	4	4	≤0.06 to 8	45.6	NA
	Levofloxacin	702	>8	>8	0.06 to >8	15.4	22.7
	Linezolid	702	2	2	≤0.5 to 8	98.2	99.9
	Minocycline	702	1	>8	≤0.03 to >8	56.8	NA
	Penicillin	702	>8	>8	≤0.06 to >8	17.4	NA
	Tetracycline	702	1	>32	≤0.06 to >32	51.4	NA
	Tigecycline	702	0.12	0.12	≤0.015 to 8	96.2^*a*^	96.2
	Vancomycin	702	1	1	≤0.12 to 4	100	100

a U.S. Food and Drug Administration (FDA) MIC interpretative susceptible breakpoints were used in place of CLSI MIC breakpoints for eravacycline (≤0.06 μg/ml) and tigecycline (≤0.25 μg/ml) (21), as none currently exist. FDA tigecycline breakpoints for vancomycin-susceptible E. faecalis were also applied to vancomycin-resistant isolates and to E. faecium.

b EUCAST breakpoints were applied for amoxicillin-clavulanate, although these are based on susceptibility testing using a fixed concentration of clavulanic acid of 2 μg/ml, while for this study, amoxicillin-clavulanate was tested with a 2:1 ratio.

c VR, vancomycin resistant; VS, vancomycin susceptible; NA, MIC breakpoint not available.

**TABLE 4:** *In vitro* activity of eravacycline and comparator agents against streptococci, cumulative 2013 to 2017 data^*g*^

Organism	Drug	No. of isolates	MIC (μg/ml)			% susceptible	
			50%	90%	Range	CLSI	EUCAST
S. pneumoniae	Eravacycline	596	0.008	0.015	≤0.001 to 0.03	NA	NA
	Amoxicillin-clavulanate	491	0.06	4	≤0.015 to >4	83.5	NA
	Azithromycin	596	0.12	>2	≤0.03 to >2	58.9	58.4
	Ceftaroline	105	0.008	0.12	≤0.004 to 0.5	100	98.1
	Ceftriaxone	596	0.03	1	≤0.015 to >2	92.3	82.4
	Clindamycin	105	0.03	>1	≤0.015 to >1	74.3	74.3
	Daptomycin	595	0.12	0.25	≤0.03 to 1	NA	NA
	Levofloxacin	596	1	1	≤0.25 to >8	99.0	99.0
	Linezolid	596	1	2	≤0.12 to 2	100	100
	Meropenem	105	≤0.03	>0.5	≤0.03 to >0.5	78.1	—
	Minocycline	596	≤0.06	8	≤0.06 to >8	NA	75.8
	Penicillin	596	≤0.12	2	≤0.12 to >2	48.0^*b*^	48.0^*b*^
	Tetracycline	596	0.12	>4	≤0.03 to >4	74.8	74.8
	Tigecycline	596	≤0.008	0.06	≤0.008 to 0.25	98.2^*a*^	NA
	Vancomycin	105	0.25	0.5	≤0.06 to 0.5	100	100
S. agalactiae	Eravacycline	1,239	0.03	0.06	0.008 to 0.25	98.0^*c*^	99.8^*d*^
	Amoxicillin-clavulanate	598	0.12	0.12	0.03 to 0.5	NA	NA
	Azithromycin	1,239	0.12	>1	≤0.03 to >2	63.7	63.6
	Ceftaroline	641	0.015	0.015	≤0.004 to 0.12	100	NA
	Ceftriaxone	1,239	0.06	0.12	≤0.015 to 0.5	100	NA
	Clindamycin	1,040	0.06	>1	≤0.015 to >1	74.5	75.9
	Daptomycin	1,239	0.25	0.5	0.06 to 1	100	100
	Levofloxacin	1,239	1	1	≤0.25 to >8	96.5	96.5
	Linezolid	1,239	1	2	0.25 to 2	100	100
	Meropenem	1,040	0.06	0.12	≤0.03 to 0.25	100	NA
	Minocycline	1,239	>8	>8	≤0.06 to >8	NA	19.9
	Penicillin	1,239	≤0.12	≤0.12	≤0.12 to 0.5	99.6	99.8
	Tetracycline	1,239	>4	>4	0.06 to >4	20.3	20.2
	Tigecycline	1,239	0.06	0.06	≤0.008 to 0.25	100^*c*^	99.8
	Vancomycin	1,040	0.5	0.5	0.25 to 1	100	100
S. pyogenes	Eravacycline	1,192	0.03	0.03	0.004 to 0.06	100^*c*^	100^*d*^
	Amoxicillin-clavulanate	665	0.03	0.03	≤0.015 to 0.25	NA	NA
	Azithromycin	1,192	0.12	0.5	≤0.03 to >2	90.1	89.9
	Ceftaroline	527	≤0.004	0.008	≤0.004 to 0.015	100	NA
	Ceftriaxone	1,192	0.03	0.03	≤0.015 to 1	99.9	NA
	Clindamycin	869	0.06	0.06	≤0.015 to >1	94.8	94.9
	Daptomycin	1,192	0.06	0.06	≤0.03 to 0.25	100	100
	Levofloxacin	1,192	0.5	1	≤0.25 to >4	99.6	99.6
	Linezolid	1,192	1	2	≤0.12 to 2	100	100
	Meropenem	869	≤0.03	≤0.03	≤0.03 to 0.12	100	NA
	Minocycline	1,192	0.12	4	≤0.06 to >8	NA	86.9
	Penicillin	1,192	≤0.12	≤0.12	≤0.12 to ≤0.12	100	100
	Tetracycline	1,192	0.25	>4	≤0.03 to >4	86.9	86.7
	Tigecycline	1,192	0.03	0.06	≤0.008 to 0.12	100^*c*^	100
	Vancomycin	869	0.5	0.5	≤0.06 to 1	100	100
S. anginosus group^*e*^	Eravacycline	346	0.03	0.06	≤0.001 to 0.12	99.1^*c*^	100
	Amoxicillin-clavulanate	138	0.06	0.25	≤0.015 to 2	NA	NA
	Azithromycin	346	0.06	>1	≤0.03 to >2	81.2	NA
	Ceftaroline	208	0.015	0.03	≤0.004 to 0.25	NA	NA
	Ceftriaxone	346	0.12	0.25	≤0.015 to >2	99.4	99.1
	Clindamycin	266	0.03	0.06	≤0.015 to >1	91.0	91.0
	Daptomycin	346	0.25	0.5	≤0.03 to 1	100	NA
	Levofloxacin	346	0.5	1	≤0.25 to >4	99.4	NA
	Linezolid	346	1	2	≤0.12 to 2	100	NA
	Meropenem	266	≤0.03	0.12	≤0.03 to 0.5	100	100
	Minocycline	346	≤0.06	8	≤0.06 to >8	NA	NA
	Penicillin	346	≤0.12	≤0.12	≤0.12 to 1	95.4	98.0
	Tetracycline	346	0.25	>4	≤0.03 to >4	69.9	NA
	Tigecycline	346	0.03	0.06	≤0.008 to 0.5	99.7^*c*^	99.1^*f*^
	Vancomycin	266	0.5	1	≤0.06 to 1	100	100

a U.S. Food and Drug Administration (FDA) MIC interpretative breakpoints were used in place of CLSI MIC breakpoints for tigecycline (≤0.06 μg/ml) (21), as none currently exist.

b Determined using the CLSI susceptible breakpoint for oral penicillin and EUCAST susceptible breakpoint for benzylpenicillin indications other than meningitis (≤0.06 μg/ml).

c U.S. Food and Drug Administration (FDA) MIC interpretative susceptible breakpoints were used in place of CLSI MIC breakpoints for eravacycline (≤0.06 μg/ml) and tigecycline (≤0.25 μg/ml) (21), as none currently exist. The FDA eravacycline susceptible breakpoint for the S. anginosus group was applied to beta-hemolytic streptococci.

d The EUCAST eravacycline susceptible breakpoint for the S. anginosus group (≤0.12 μg/ml) was applied to beta-hemolytic streptococci.

e The S. anginosus group (*n* = 346) includes S. anginosus (*n* = 302), S. constellatus (*n* = 36), S. intermedius (*n* = 7), and S. intermedius/S. milleri (*n* = 1).

f EUCAST tigecycline breakpoints for beta-hemolytic streptococci (≤0.12 μg/ml) were applied to the S. anginosus group.

g NA, MIC breakpoint not available; —, not evaluable, as the tested MIC range did not extend high enough for the EUCAST susceptible breakpoint for S. pneumoniae.

When isolates were allocated to their respective geographic regions, eravacycline MIC_90_s were within 1 doubling dilution for all Gram-positive genera/species (see Table S3 in the supplemental material). Similarly, there were no significant differences (a >1-doubling-dilution increase or decrease in MIC_90_s) observed in the *in vitro* activity of eravacycline for any genera/species of Gram-positive bacteria stratified by study period (2013 to 2014, 2015, 2016, 2017) (Table S4) or stratified by specimen source (Table S5). A detailed trend analysis could not be conducted, given that there were changes in participating laboratories and the panel of antimicrobial agents tested over the time period studied (2013 to 2017). Overall, eravacycline activity was similar over time and across geographic regions and specimen sources.

Eravacycline consistently demonstrated 2- to 4-fold lower MIC_90_ values than tigecycline for populations of Gram-positive pathogens. Previous *in vitro* studies comparing eravacycline and tigecycline have reported similar 2- to 4-fold improvements in the MIC_90_ (4, 6, 7, 15). Susceptibility rates, due to a difference in breakpoints, were similar between these two antibiotics. As tigecycline EUCAST breakpoints have recently been lowered for Gram-negative organisms, perhaps a review of the breakpoints for Gram-positive organisms is also warranted for this agent.

This global surveillance investigation highlights the broad-spectrum potency of eravacycline against Gram-positive bacteria, including resistant isolates. As cIAIs are well-known to be polymicrobial, involving synergistic Gram-positive, Gram-negative, and anaerobic organism interactions, this study underscores the potential benefit of eravacycline for the empirical treatment of cIAIs. Furthermore, eravacycline may have a role in the treatment of other infections caused predominantly by Gram-positive pathogens, but the clinical utility in such disease states should be investigated.

## MATERIALS AND METHODS

### Bacterial isolates.

From 2013 to 2017, 10,511 clinical isolates of *Enterococcus* spp. (*n* = 2,807), *Staphylococcus* spp. (*n* = 4,331), and *Streptococcus* spp. (*n* = 3,373) were collected by laboratories in 37 countries on three continents (Asia/Pacific, Europe, North America). The identity of each isolate was confirmed using matrix-assisted laser desorption ionization–time of flight (MALDI-TOF) mass spectrometry (Bruker Biotyper; Bruker Daltonics, Bremen, Germany).

Table S1 in the supplemental material summarizes the numbers of isolates collected in each of the four study periods (2013 to 2014, 2015, 2016, and 2017) by geographic region. Overall, approximately 54% of the isolates came from Europe, 35% of the isolates came from North America, and 10% came from the Asia-Pacific region. In total, there were 3,180, 2,082, 3,176, 956, and 1,117 isolates, respectively, from respiratory, intra-abdominal, urinary, skin, and other specimen sources (Table S2).

Isolates were limited to one per patient, determined by the participating laboratory algorithms to be clinically significant, and collected irrespective of their antimicrobial susceptibility profile and independent of patient gender or age. The study was not designed to directly compare the prevalence of antimicrobial-resistant pathogens across specific geographic locations but, rather, was designed to evaluate the *in vitro* activities of eravacycline and the comparator antimicrobial agents against a global collection of frequently encountered clinical isolates of Gram-positive bacteria collected from 2013 to 2017.

### Antimicrobial susceptibility testing.

The *in vitro* susceptibilities of the isolates were determined using the CLSI-defined broth microdilution method in 96-well broth microdilution panels (18, 19). The antimicrobial agents used in panel production were acquired as laboratory-grade powders from their respective manufacturers or from a commercial source. The list of antimicrobial agents tested in each of the four study periods varied slightly, in that some agents, in addition to those tested in the 2013 to 2014 period, were included in the 2015, 2016, and 2017 testing periods. Of note, ampicillin, clindamycin, meropenem, and oxacillin were tested only in 2015, 2016, and 2017. The eravacycline MICs for Gram-positive bacteria were read following the current CLSI standard for dilution method testing; MIC endpoints were read following panel incubation at 35°C in ambient air for 16 to 20 h (*Enterococcus* and *Staphylococcus* spp.) or 35°C in ambient air for 20 to 24 h (*Streptococcus* spp.) (19). Quality control testing for eravacycline and the other antimicrobial agents was performed on each day of testing, as specified by the CLSI, using the CLSI-defined control strains E. faecalis ATCC 29212, S. aureus ATCC 29213, and S. pneumoniae ATCC 49619 (19).

MICs were interpreted using 2019 CLSI MIC breakpoints (19) and 2019 EUCAST MIC breakpoints (20), with the following exceptions. FDA MIC interpretative breakpoints were used for tigecycline (21) and eravacycline in place of CLSI MIC breakpoints, which are not currently published for these agents. Additionally, tigecycline breakpoints for vancomycin-susceptible Enterococcus faecalis were applied to vancomycin-resistant isolates and to Enterococcus faecium; EUCAST eravacycline breakpoints for the Streptococcus anginosus group were applied to beta-hemolytic streptococci; EUCAST tigecycline breakpoints for beta-hemolytic streptococci were applied to the S. anginosus group; and EUCAST eravacycline breakpoints for S. aureus were applied to coagulase-negative *Staphylococcus* species.

## Supplementary Material

Supplemental file 1
