# Supplementary material for: In Vitro Activity of Eravacycline against Gram-Positive Bacteria Isolated in Clinical Laboratories Worldwide from 2013 to 2017
Source: Antimicrob Agents Chemother. 2020 Feb 21;64(3):e01715-19. doi: 10.1128/AAC.01715-19 (PMC7038300; doi:10.1128/AAC.01715-19)
Supplement: Supplemental file 1 [file AAC.01715-19-s0001.pdf]

SUPPLEMENTAL TABLE 1 Distribution of clinical isolates of staphylococci, enterococci, and streptococci by region, country and study period

Region	Country	Study period/number of isolates				
		2013-2014	2015	2016	2017	Total
Asia/Pacific	Australia		25	12	47	84
	Hong Kong		20	0	0	20
	Japan		20	64	26	110
	South Korea		64	44	113	221
	Malaysia		30	1	5	36
	Pakistan		13	6	2	21
	Philippines		41	65	90	196
	Singapore		16		0	16
	Taiwan		77	50	92	219
	Thailand		47	51	52	150
	Vietnam		0	6	12	18
Europe	Austria	5	3	31		39
	Belgium	4	132	144	107	387
	Croatia		28	31	18	77
	Czech Republic	32	54	46	62	194
	Denmark	4	23	15	7	49
	France	140	221	158	265	784
	Germany	143	174	162	173	652
	Greece	102	48	18	84	252
	Hungary	35	45	54	60	194
	Ireland	11	12	4	13	40
	Italy	137	215	135	218	705
	Latvia			13	7	20
	Lithuania			20	14	34
	Netherlands	17	62	52	47	178
	Poland	107	18	16	30	171
	Portugal	45	73	49	58	225
	Romania	104	7	20	1	132
	Russia	108	29	41	32	210
	Serbia				8	8
	Spain	143	199	118	220	680
	Sweden	16			2	18
	Switzerland		18	28	55	101
	Turkey	105	63	103	47	318
	United Kingdom	101	44	25	65	235
North America	Canada			17		17
	United States	2108	519	344	729	3700
Total		3467	2340	1943	2761	10511

SUPPLEMENTAL TABLE 2 Distribution of clinical isolates of staphylococci, enterococci, and streptococci by specimen source and study period

	Study period/number of isolates				
Specimen source	2013-2014	2015	2016	2017	Total
Intra-abdominal					
Staphylococci	236	161	191	346	934
Enterococci	294	89	123	183	689
Streptococci	86	91	84	198	459
Respiratory					
Staphylococci	240	547	444	415	1646
Enterococci	23	42	40	65	170
Streptococci	515	220	276	353	1364
Urinary					
Staphylococci	162	177	273	361	973
Enterococci	337	247	243	524	1351
Streptococci	87	199	257	309	852
Skin					
Staphylococci	574				574
Enterococci	106				106
Streptococci	276				276
Other					
Staphylococci	202	1	1		204
Enterococci	200	277	8	6	491
Streptococci	129	289	3	1	422
Total	3467	2340	1943	2761	10511

SUPPLEMENTAL TABLE 3 *In vitro* activity of eravacycline and selected comparator agents against staphylococci, enterococci, and streptococci stratified by region

			µg/ml									
			Eravacycline		Tigecycline		Tetracycline		Minocycline		Linezolid	
Organism	Region	*n*	MIC_{50}	MIC_{90}	MIC_{50}	MIC_{90}	MIC_{50}	MIC_{90}	MIC_{50}	MIC_{90}	MIC_{50}	MIC_{90}
S. aureus	Asia/Pacific	346	0.06	0.25	0.12	0.5	0.25	>16	0.12	8	1	2
	Europe	926	0.06	0.12	0.12	0.25	0.25	>16	0.12	0.25	2	2
	North America	1316	0.06	0.12	0.12	0.25	0.25	1	0.12	0.25	2	2
S. epidermidis	Asia/Pacific	89	0.12	0.5	0.12	0.25	1	>16	0.12	0.5	≤0.5	1
	Europe	656	0.12	0.5	0.12	0.5	1	16	0.12	0.5	≤0.5	2
	North America	267	0.12	0.5	0.25	0.5	1	>16	0.25	0.5	1	2
S. haemolyticus	Asia/Pacific	74	0.12	0.5	0.25	0.5	1	>16	0.25	0.5	1	1
	Europe	607	0.12	0.25	0.25	0.5	1	>16	0.12	0.25	1	2
	North America	50	0.03	0.5	0.12	1	0.25	>16	≤0.06	0.5	1	2
E. faecalis	Asia/Pacific	137	0.06	0.06	0.12	0.25	>32	>32	>8	>8	1	2
	Europe	802	0.06	0.06	0.12	0.25	>32	>32	8	>8	1	2
	North America	647	0.06	0.06	0.12	0.25	>32	>32	>8	>8	2	2
E. faecium	Asia/Pacific	95	0.03	0.06	0.12	0.12	>32	>32	8	>8	1	2
	Europe	617	0.03	0.06	0.06	0.25	0.5	>32	1	>8	1	2
	North America	509	0.03	0.06	0.12	0.25	32	>32	8	>8	2	2
S. pneumoniae	Asia/Pacific	20	0.015	0.03	0.06	0.12	>4	>4	8	>8	1	2
	Europe	202	0.008	0.015	≤0.008	0.06	0.12	>4	≤0.06	8	1	2
	North America	374	0.008	0.015	≤0.008	0.03	0.12	>4	≤0.06	8	1	2
S. agalactiae	Asia/Pacific	178	0.03	0.06	0.06	0.06	>4	>4	>8	>8	1	2
	Europe	830	0.03	0.06	0.06	0.06	>4	>4	>8	>8	1	2
	North America	231	0.03	0.06	0.06	0.06	>4	>4	>8	>8	1	2
S. pyogenes	Asia/Pacific	104	0.03	0.06	0.06	0.06	0.25	>4	0.12	>8	1	1
	Europe	840	0.03	0.03	0.03	0.06	0.25	4	≤0.06	1	1	2
	North America	248	0.015	0.03	0.03	0.06	0.12	>4	0.12	4	1	2
S. anginosus group	Asia/Pacific	48	0.03	0.06	0.03	0.12	2	>4	0.5	>8	1	2
	Europe	223	0.03	0.06	0.03	0.06	0.25	>4	≤0.06	8	1	2
	North America	75	0.015	0.03	0.03	0.06	0.25	>4	≤0.06	8	1	2

SUPPLEMENTAL TABLE 4 *In vitro* activity of eravacycline and selected comparator agents against staphylococci, enterococci, and streptococci stratified by study period

			µg/ml									
			Eravacycline		Tigecycline		Tetracycline		Minocycline		Linezolid	
Organism	Study period	*n*	MIC_{50}	MIC_{90}	MIC_{50}	MIC_{90}	MIC_{50}	MIC_{90}	MIC_{50}	MIC_{90}	MIC_{50}	MIC_{90}
S. aureus	2013-2014	980	0.06	0.12	0.12	0.25	0.5	8	0.12	0.25	2	2
	2015	532	0.06	0.06	0.12	0.25	0.25	16	0.12	0.25	1	2
	2016	512	0.06	0.12	0.25	0.25	0.25	>16	≤0.06	0.12	1	2
	2017	564	0.03	0.06	0.12	0.25	0.25	>16	≤0.06	2	1	2
S. epidermidis	2013-2014	277	0.25	0.5	0.25	0.5	2	>16	0.25	0.5	1	2
	2015	206	0.12	0.25	0.12	0.25	1	16	0.25	0.5	≤0.5	1
	2016	221	0.12	0.5	0.12	0.5	1	>16	0.12	0.25	≤0.5	1
	2017	308	0.06	0.25	0.12	0.25	1	8	0.12	0.25	≤0.5	1
S. haemolyticus	2013-2014	157	0.12	0.5	0.25	0.5	1	>16	0.25	0.5	1	2
	2015	148	0.12	0.5	0.25	0.5	1	>16	0.25	0.25	1	1
	2016	176	0.12	0.25	0.25	0.5	1	>16	0.12	0.25	≤0.5	1
	2017	250	0.03	0.25	0.12	0.5	0.5	>16	≤0.06	0.25	1	1
E. faecalis	2013-2014	501	0.06	0.06	0.12	0.25	32	>32	8	>8	2	2
	2015	418	0.06	0.06	0.12	0.25	>32	>32	8	>8	1	1
	2016	258	0.06	0.06	0.12	1	>32	>32	>8	>8	1	2
	2017	409	0.06	0.06	0.12	0.25	>32	>32	>8	>8	2	2
E. faecium	2013-2014	459	0.06	0.06	0.12	0.25	32	>32	8	>8	2	2
	2015	237	0.03	0.06	0.06	0.12	2	>32	0.25	>8	1	2
	2016	156	0.03	0.06	0.12	0.5	0.5	>32	0.5	>8	1	2
	2017	369	0.03	0.06	0.06	0.12	32	>32	8	>8	2	2
S. pneumoniae	2013-2014	491	0.008	0.015	≤0.008	0.015	0.12	>4	≤0.06	8	1	2
	2015	0	--	--	--	--	--	--	--	--	--	--
	2016	0	--	--	--	--	--	--	--	--	--	--
	2017	105	0.015	0.03	0.06	0.06	0.25	>4	≤0.06	>8	1	2
S. agalactiae	2013-2014	199	0.015	0.03	0.03	0.03	>4	>4	>8	>8	2	2
	2015	399	0.06	0.06	0.06	0.12	>4	>4	>8	>8	1	1
	2016	304	0.03	0.06	0.06	0.06	>4	>4	>8	>8	1	2
	2017	337	0.03	0.06	0.06	0.06	>4	>4	>8	>8	1	2
S. pyogenes	2013-2014	323	0.015	0.015	0.015	0.03	0.12	>4	0.12	4	1	2
	2015	342	0.03	0.03	0.06	0.06	0.25	>4	0.12	4	1	1
	2016	258	0.015	0.03	0.06	0.06	0.25	>4	≤0.06	2	1	1
	2017	269	0.03	0.03	0.06	0.06	0.25	>4	≤0.06	4	1	1
S. anginosus group	2013-2014	80	0.008	0.03	≤0.008	≤0.008	0.12	>4	≤0.06	4	1	2
	2015	58	0.03	0.06	0.03	0.06	0.5	>4	0.12	>8	1	2
	2016	58	0.015	0.03	0.03	0.06	0.5	>4	≤0.06	>8	1	1
	2017	150	0.03	0.06	0.06	0.06	0.5	>4	≤0.06	>8	1	2

SUPPLEMENTAL TABLE 5 *In vitro* activity of eravacycline and selected comparator agents against staphylococci, enterococci, and streptococci stratified by specimen source

			μg/ml									
			Eravacycline		Tigecycline		Tetracycline		Minocycline		Linezolid	
Organism	Specimen source	*n*	MIC_{50}	MIC_{90}	MIC_{50}	MIC_{90}	MIC_{50}	MIC_{90}	MIC_{50}	MIC_{90}	MIC_{50}	MIC_{90}
S. aureus	Intra-abdominal	436	0.06	0.12	0.12	0.25	0.25	>16	0.12	0.25	2	2
	Respiratory	1,212	0.06	0.12	0.12	0.25	0.25	>16	0.12	0.25	1	2
	Urinary	338	0.06	0.12	0.12	0.25	0.25	>16	≤0.06	0.25	1	2
	Skin	431	0.06	0.12	0.12	0.25	0.5	16	0.12	0.25	2	2
S. epidermidis	Intra-abdominal	353	0.12	0.25	0.12	0.5	1	16	0.12	0.5	1	1
	Respiratory	234	0.12	0.5	0.12	0.25	1	16	0.12	0.5	≤0.5	1
	Urinary	287	0.12	0.5	0.12	0.5	1	16	0.12	0.5	≤0.5	2
	Skin	120	0.25	0.5	0.25	1	2	>16	0.25	0.5	2	2
S. haemolyticus	Intra-abdominal	145	0.12	0.25	0.25	0.5	1	>16	0.12	0.25	1	1
	Respiratory	200	0.12	0.5	0.25	0.5	1	>16	0.25	0.5	1	1
	Urinary	348	0.06	0.25	0.12	0.5	0.5	>16	0.12	0.25	1	2
	Skin	23	0.25	0.5	0.25	1	1	>16	0.25	0.5	1	2
E. faecalis	Intra-abdominal	277	0.06	0.06	0.12	0.25	32	>32	8	>8	2	2
	Respiratory	90	0.06	0.06	0.12	0.25	>32	>32	>8	>8	1	2
	Urinary	897	0.06	0.06	0.12	0.25	>32	>32	>8	>8	1	2
	Skin	58	0.06	0.06	0.12	0.25	>32	>32	>8	>8	2	2
E. faecium	Intra-abdominal	412	0.03	0.06	0.12	0.25	0.5	>32	1	>8	2	2
	Respiratory	80	0.03	0.06	0.12	0.25	32	>32	8	>8	1	2
	Urinary	454	0.03	0.06	0.12	0.12	32	>32	8	>8	2	2
	Skin	48	0.06	0.06	0.12	0.25	>32	>32	>8	>8	2	2
S. pneumoniae	Intra-abdominal	46	0.015	0.015	0.03	0.06	0.12	>4	≤0.06	>8	1	2
	Respiratory	500	0.008	0.015	≤0.008	0.03	0.12	>4	≤0.06	8	1	2
	Urinary	18	0.015	0.015	0.06	0.06	0.25	>4	≤0.06	>8	1	1
	Skin	14	0.008	0.008	≤0.008	0.015	0.12	>4	0.12	8	1	2
S. agalactiae	Intra-abdominal	124	0.03	0.06	0.06	0.12	>4	>4	>8	>8	1	2
	Respiratory	156	0.03	0.06	0.06	0.06	>4	>4	>8	>8	1	2
	Urinary	720	0.03	0.06	0.06	0.06	>4	>4	>8	>8	1	2
	Skin	19	0.015	0.06	0.03	0.03	>4	>4	>8	>8	1	2
S. pyogenes	Intra-abdominal	86	0.03	0.03	0.03	0.06	0.25	>4	≤0.06	2	1	1
	Respiratory	644	0.03	0.03	0.06	0.06	0.25	>4	≤0.06	4	1	2
	Urinary	77	0.015	0.03	0.03	0.06	0.12	0.25	≤0.06	0.12	1	2
	Skin	216	0.015	0.015	0.015	0.03	0.12	>4	0.12	8	1	2
S. anginosus group	Intra-abdominal	203	0.03	0.06	0.03	0.06	0.25	>4	≤0.06	8	1	2
	Respiratory	64	0.03	0.06	0.03	0.06	0.25	>4	≤0.06	>8	1	2
	Urinary	37	0.015	0.03	0.03	0.06	>4	>4	1	8	1	2
	Skin	27	0.008	0.03	≤0.008	≤0.008	0.12	8	≤0.03	8	1	2
